# Magnitude of depression and associated risk factors among patients with musculoskeletal disorder treated in physiotherapy outpatient department in Amhara region comprehensive specialized hospital in Ethiopia: a prospective cross-sectional study

**DOI:** 10.1186/s12888-023-04658-3

**Published:** 2023-03-22

**Authors:** Ermias Solomon Yalew, Ashenafi Zemed Melese, Yisak Girma Guadie, Yohannes Abich, Tesfa Kassa, Moges Gashaw

**Affiliations:** Department of Physiotherapy, School of Medicine, College of Medicine and Health Sciences, University of Gondar Comprehensive Specialized Hospital, P.O. Box: 196, Gondar, Ethiopia

**Keywords:** Musculoskeletal disorder, Depression, Patient health questionnaire, Ethiopia

## Abstract

**Background:**

The state of depression that can lead to substance and drug abuse, as well as an increased risk of suicide. Without a doubt, the link between musculoskeletal pain and depression compromises a person’s health and drastically lowers their quality of life, resulting in increased incapacity. Depression and musculoskeletal discomfort are two well-known risk factors for long-term sickness absence, which is defined as a period of sickness lasting more than a week, which means chronic musculoskeletal pains, particularly multiple pains, are linked to depression. And early diagnosis and care of depression in musculoskeletal disorder are critical to patients’ physical, functional, and occupational results. This study aimed to assess the magnitude of depression and associated risk factors among patients with musculoskeletal disorder.

**Method:**

Multi-institution cross-sectional study conducted in Amhara region Comprehensive specialized Hospitals from April 1st to May 30th, 2021. The data was collected from 217 participants through interview administrative questioner and patient medical record review. Binary logistic regression was used to identify associated risk factors of depression. The strength of the association was detected by the adjusted odds ratio.

**Result:**

A total of 217 participate in this study with the age range of 18–80 years. Among the study participants, 57.1% (n = 124) (AOR: 95% CI: 50.7–63.6) were had depression. Treatment duration, and social support were significantly association with depression among patient with musculoskeletal disorder with P < 0.05.

**Conclusion:**

The magnitude of depression was relatively high among musculoskeletal disorder patients treated in physiotherapy out-patient department. The length of treatment duration (hospital stay) and the status of social support from families and friends were significantly associated with depression among patients with musculoskeletal disorders. A multidisciplinary strategy is required for diagnosing and treating depression in patients with musculoskeletal disorder.

**Supplementary Information:**

The online version contains supplementary material available at 10.1186/s12888-023-04658-3.

## Background

Depression is a major public health problem and the leading disease burden among all mental and physical illnesses, accounting for 10% of total years lived with disability (YLD) in Low- and Middle-Income Countries (LMICs) [1]. Depression is a mood disorder characterized by sadness, loss of interest or pleasure, feelings of guilt or low self-worth, disturbed sleep and appetite, feelings of tiredness, lowering of mood and poor concentration [2] .

Based on the World Health Organization (WHO) report, globally around 350 million people are suffering from depression [3]. According to global mental health survey, the prevalence of depression in high income countries is 15% [4]; in low and middle-income countries (LMIC), it ranges from 11.1 to 53% [2]. However, the magnitude is different from place to place in the world; for instance, in the western Pacific, the estimate is 3.6% and becomes 5.4% in the Africa region [4]. In Ethiopia, depression is among the top-ten high burden diseases [5]. Based on National Health Survey data, conducted in Ethiopia, the prevalence of depression in the general population was 9.1% [6]. However, the burden of depression increases among people with chronic conditions and associated comorbidity; Without a doubt, the link between musculoskeletal pain and depression compromises a person’s health and drastically lowers their quality of life [7].

Musculoskeletal disorder (MSD) is the major source of disability, work absenteeism and psychological distress [8]. Musculoskeletal (MSK) pain is a major public health issue, with a global prevalence range of 11.4–60% [9]. It is the leading cause of both physical and occupational disability [10]. MSK pain has a major impact on people’s quality of life [11]. MSD is a broad term for a variety of inflammatory, degenerative diseases and disorders of musculoskeletal system that cause pain and functional impairments in people exposed to work activities, as well as conditions that contributed to the development or exacerbation of the condition but were not the sole cause [12].

Depression and musculoskeletal discomfort are two well-known risk factors for long-term sickness absence [13]. People who are depressed frequently report pain and depression. On the other hand, depressive symptoms are common in chronic pain patients and depression can lead chronic pain [14]. Depression may be caused by pain and the constraints it imposes on the patient’s life, but chronic pain may also be a symptom of an underlying depressive disorder, shown through the symptom of pain [15].

A systematic review done by using eight article conclude that the pool prevalence of depression was 20% [16]. The national wide observational study done in United State from 2011 to 2015 revealed that 22.9% of hospitalized patient had comorbid depression [17]. The study conducted in Korea showed that 35.1% patients had unrecognized depressive symptoms [18]. The study done in Saudi Arabia revealed that the prevalence of depression was 71% [19]. In African study done in Nigeria Yobe State Specialist Hospital showed that 65.3% individual had depression [20]. Similarly the study done in Nigeria 46.1% patients was experienced depression among stroke survivor [21]. The study done in orthopedic trauma patient of Ethiopia, the depressive symptoms was ranged from 36.1 to 36.4% [22, 23].

Previous epidemiological studies revealed that female gender, older age, having comorbidities, a longer hospital stay, educational status, severity of pain, location of pain, and social support had significantly associated with depression among people with musculoskeletal pain [17–19, 23, 24]. Musculoskeletal disorder in particular is often comorbid with depression, a leading cause of disability and increase the risk of depression[23]. There is enough evidence on the burden of depression in the general population however the magnitude of depression among musculoskeletal disorder patients is not routinely assessed. Therefore, the aim of this study is to assess the burden of depression and its associated factors among patients with musculoskeletal disorders in Ethiopia.

## Methods and materials

### Study design and setting

An institution based cross-sectional study was conducted from April 1st to May 30th, 2021 in Amhara regional state Comprehensive Specialized Hospitals namely university of Gondar, Felege Hiwot, and Tsbebe Ghion comprehensive specialized hospitals.

The University of Gondar and Tsbebe Ghion Comprehensive Specialized Teaching Hospitals are the two-university hospital that give comprehensive physiotherapy services and taught physiotherapy program in under and postgraduate level in the region. In addition, Felege Hiwot comprehensive specialized hospital give comprehensive physiotherapy service for a large group of population. All three hospitals which the data was collected have similar clinical setup and give similar physiotherapy service for the musculoskeletal disorder patients.

### Source population, study population, inclusion and exclusion criteria

Adult with musculoskeletal pain who were treated in outpatient physiotherapy department were study’s source population. The study population were all adult with musculoskeletal disorder (MSD) who were treated under outpatient department of physiotherapy at Gondar and Bahir Dar comprehensive specialized hospitals during the data collection period. Individuals with musculoskeletal disorder who had at least a two-week follow-up in the outpatient physiotherapy department and their age above 18 years old were included in the study. MSD patients who had been receiving physiotherapy for less than two weeks, as well as those with memory, comprehension, communication deficits, individual with psychological disorders based on the finding from the patient’s medical registration chart were excluded from the study.

### Sample size and sampling procedure

The sample size was determined and calculated by using single population proportion formula; 95% confidence interval, 0.5 prevalence since there is no previous study done in depression among musculoskeletal disorder in Ethiopia, 5% precision. The sample size was estimated as follows:

n = (za/2) ² p (1-p)/d2.

n= (1.96)2* (0.5) * (0.5)/ (0.05) ² = 385.

n = 385 However the samples were obtained from a relatively small population (N = 600); correction formula was used.

n = n/1 + n/N.

n = 385/1 + 385/600.

The derived power calculated sample size was n = 235.

To ensure representativeness, proportional allocation was done for each comprehensive specialized hospitals 117, 78, 40 participants were selected from UOGCSH, FHCSH and TGCSH. Systematic random sampling was employed to enumerate the study subjects, “K” was calculated to be 2, (N = averagely expected number of patients come to hospital per two month 600, n = 235; K = 600/235 = 2). Lottery method used to draw the 1st sample of the first 2 participants and continues with every Kth interval for each outpatient physiotherapy department.

### Data collection instrument

An interviewer-administered questioner was prepared from the literature and patient medical record review was done to gather information. The Amharic version of the Patient Health Questionnaire (PHQ-9) was used to measure the dependent variable of depression (yes/no). Which has been validated in Ethiopia with a sensitivity of 86%.

PHQ-9 is a versatile questionnaire used for depression screening, diagnosis, monitoring, and severity assessment. It had 9-item for depression, and the maximum score is 27 on a three-point severity scale over the last two weeks preceding the survey. Based on the instrument standard, a PHQ-9 score ≥ 10 is considered significant for meeting any form of potential depression diagnosed as a disorder Participants were considered to have depression and PHQ ≤ 10 patcipants were consider as potential no depression [25].

Independent variables like, socio-demographic, and clinical characteristics: physiotherapy diagnosis, co-morbidity, duration of treatment, and pain (pain intensity was measured bay Visual Analogue Scale (VAS) 0 = No pain, 1–3 = mild pain, 4–6 = moderate, 7–10 = Intense pain) [26] was collected using an interviewer administered questionnaires and that were prepared in Amharic.

Musculoskeletal disorder (MSKD) is if the patient experiences a complaint of unpleasant sensation(pain), ache, discomfort interfering with their activity of daily life at any part of their body region( neck, shoulder, elbow, wrist, back, hip, knee, ankle and foot) at any time during the last twelve months[27].

Social support was measured by the Oslo-3 scale has been used in several studies, thus confirming its feasibility and predictive validity with respect to psychological distress to state the prevalence of social support, we use the sum score scale ranging from 3–14(poor = 3–8, moderate = 9–11, and strong = 12–14) [28] (Additional file 1).

### Data quality assurance

The data was gathered through pre-tested face-to-face interviews with a questioner and a review of patient medical records. The data was collected by three experienced musculoskeletal physiotherapists. The data collectors received two days of intense instruction from the principal investigator (ES) and MG on how to approach study participants, how to use the questionnaire and guidelines, and data collection procedures. The investigators kept a close eye on the data collection technique and evaluated the obtained questionnaire for accuracy, completeness, and consistency on a daily basis.

### Data processing and analysis

The data was coded and entered using Epi-Data version 4.6.0.4 before being exported to SPSS for Windows version 20. Investigators cleared the data and carefully verified it for missing numbers. Socio-demographic data was interpreted using descriptive statistical analysis. To investigate the associations between dependent and independent variables, binary logistic regression was used. To control for potential confounders, univariable independent variables with p-values of 0.2 were transferred to multivariable logistic regression analysis. The strength of associations was determined using an adjusted odds ratio (AOR) with a 95% confidence interval (CI) with a p-value of less than 0.05. The model fitting was checked by the Hosmer-Lemeshow goodness of fit test which had a result of (p = 0.586).

## Result

### Socio-demographic characteristics of the participant

A total of two hundred seventeen participants were included in this study, with a respondent rate of 92.3%. The reason for non-respond was unwilling to participate, and refuse to take consent. The age of the study participants was ranged from 18 to 80 years with the mean age of (37.67 ± 11.895 years). From the total participants, more than half (52.5%) of the participant were male, more than two-third (71.4%) of the participants have been living in the urban area. More than one-third (38.2%) of the participants were government, nearly one-third (34.1%) of the participants were completed collage and above education (Table 1)

**Table 1:** Socio-demographic characteristics of patients with musculoskeletal disorders in out-patient physiotherapy department at Amhara compressive specialized hospitals, Ethiopia (n = 217)

Variables	Categories	Frequency(n)	Percentage (%)
Age in years (Mean age (37.67 ± 11.895)	18–35	107	49.3
	36–55	32	39.8
	56–80	28	12.9
Residence	Urban	155	71.4
	Rural	62	28.6
Gender	Male	114	52.5
	Female	103	47.5
Marital status	Married	136	62.7
	Single	60	27.6
	divorced	21	9.7
Religion	Orthodox Christian	166	76.5
	Muslims	26	12
	protestant	25	11.5
Occupation	Government employed	83	38.2
	Self- employed	61	28.1
	House wife	28	12.9
	Unemployed	24	11.1
	Farmer	21	9.7
Level of education	Not able to read and write	39	18
	Able to read and write	16	7.4
	Primary school	28	12.9
	Secondary school	60	27.6
	College and above	74	34.1

### Clinical characteristics and social support of the participants

Nearly half (47.7%) of the participants had between 3 and 4 weeks of treatment, and the majority (88.9%) of the participants were complaining of pain; among them, nearly two-thirds (64.9%) of the participants had mild pain. From the total, 51 participants had co-morbidity (another medical condition); among them, one-third (35.2) of the participants had DM (Table 2).

**Table 2:** Clinical characteristics of patients with musculoskeletal disorders in out-patient physiotherapy department at Amhara compressive specialized hospitals, Ethiopia (n = 217)

Variables categories		Frequency	Percent %
Treatment duration	2 week	45	20.7
	3–4 weeks	104	47.9
	> 4 weeks	68	31.3
Pain	Yes	193	88.9
	No	24	11.1
Pain severity	Mild pain	141	64.9
	Moderate Pain	52	24.0
	Severe pain	0	0.0
Having comorbidity	Yes	51	23.5
	No	166	76.5
Types of comorbidity	Hypertension	11	21.5
	DM	18	35.2
	Hypertension and DM	3	5.8
	asthma	5	9.8
	kidney disease	5	9.8
	RVI	4	7.8
	nerve injury	5	9.8
Social support	Poor	50	23
	Moderate	93	42.9
	Strong	74	34.1

### Prevalence of depression among musculoskeletal patients

In the present study, 141 patients had potential depression, accounting for a prevalence of 57.1% (95% CI: 50.7–63.6). Among the musculoskeletal patients who had potential depression, 54% (n = 67) were male, whereas 46% (n = 57) were female. The proportion of potential depression was higher in urban resident 75% (n = 93) and most of the patients were experienced mild pain 67.7%(n = 84).

### Factors associated with depression among musculoskeletal patient

A binary logistic regression analysis was performed on each of the independent factors that were found to be significantly associated with the prevalence of potential depression among musculoskeletal patients. Age, address, severity of pain, having co-morbidity, treatment duration, and social support were significantly associated with the prevalence of potential depression among musculoskeletal patients at a P-value < 0.2 in the bi-variable logistic regression. Those variables were moved into the multivariable logistic regression and further analyzed to adjust for potential confounders and identify predictors of the prevalence of potential depression among musculoskeletal patients. In a multivariable logistic regression analysis, treatment duration and social support were variables significantly associated with the prevalence of potential depression among musculoskeletal patients.

Patients with treatment durations of 3 to 4 weeks had 2.354 times the odds of being potentially depressed as patients with treatment durations of only 2 weeks (AOR: 2.354, 95% CI (1.040–5.327)). Individuals who received treatment for more than four weeks had a 3.20-fold increased risk of developing potential depression compared to musculoskeletal patients who received treatment for two weeks (AOR: 3.206; 95% CI (1.328–7.738)). The model also showed that moderate social support musculoskeletal patients were 2.812 times more likely (AOR: 2.812; 95% CI: 1.401–5.643) and those with poor social support were 10.32 times more likely (AOR: 10.319; 95% CI: 3.964–26.863) to have potential depression as compared to those with strong social support (Table 3).

**Table 3:** Bi-variable and multivariable logistic regression analysis on factors associated with depression among patients with musculoskeletal disorders at Amhara compressive specialized hospitals, Ethiopia (n = 217)

Variables	Depression		Univariate COR (95%CI)	Multivariate AOR (95%CI)	P value
	No	Yes			
Age in years					
18–35	65	65	1		
36–55	35	47	0.868(0.483–1.557)	1.422(0.526–3.846)	0.488
56–80	16	12	0.485(0.209–1.126)	1.766(0.657–4.758)	0.260
**Residence**					
Urban	62	93	1.500(0.829–2.713)	1.528(0.774–3.020)	0.222
Rural	31	31	1		
**Having comorbidity**					
Yes	28	23	0.529(0.281–0.996)	0.628(0.304–1.298)	0.209
No	65	101	1		
**Treatment duration**					
2 week	30	15	1		
3–4 weeks	41	63	3.073(1.475–6.403)	2.354(1.040–5.327)	0.040*
> 4 weeks	22	46	4.182(1.876–9.320)	3.206(1.328–7.738)	0.010*
**Social support**					
Poor	7	43	11.341(4.472–28.758)	10.319(3.964–26.863)	0.000***
Moderate	38	55	2.672(1.421–5.024)	2.812(1.401–5.643)	0.004**
Strong	48	26	1		

1 = reference category AOR = Adjusted odds ratio, CI = confidence interval, COR = crudes odds ratio, *=statistically significant at p < 0.05

## Discussion

The aim of this study was to estimate the burden of depression among patients with musculoskeletal disorders in the physiotherapy outpatient department at the comprehensive specialized hospital in the Amhara region. The overall burden of depression among patients with musculoskeletal disorders in the physiotherapy outpatient department was 57.1% (95% CI: 50.7–63.6), and treatment duration and social support were significantly associated with depression among patients with musculoskeletal disorders.

The finding of the present study is comparable with the study done in India among patients with mechanical low back pain: 52% [29]; 60.8% in patients attending pain specialized treatment centers in London, United Kingdom [30]; and 59.3% in patients attending seven hospitals in China [31].

In contrast, the magnitude of depression is larger than the findings from studies conducted in the USA: 22.3% among musculoskeletal pain disorders in an outpatient rehabilitation program [32]. This difference may be due to methodology, USA study used community-based survey but our study used systematic random sampling and proportional allocation is incorporated. Similarly, a systematic study done by using forty studies in UK revealed that 19.9% pooled prevalence of depression in osteoarthritis [33]. The possible justification for this difference may be the difference in the population reviewed; the above review included specifically osteoarthritis patients, but the present study included a patient with osteoarthritis and other musculoskeletal conditions.

Furthermore, it was detected the result of this study was detected at much higher in magnitude than the study done in Greece 22. 5% [34] and 26% in UK among patients with chronic pain [35]. The possible justification for this inconsistency might be due to the study population or setting. In this study, we included a patient with any type of musculoskeletal disorder visiting an outpatient physiotherapy department, whereas the studies in Greece and the UK used any patient who had any chronic pain; 28.2% of patients in China among patients with frozen shoulder [36]. This difference might be the variation of the study population this study conducts all patients with musculoskeletal disorders but the China study conducts by using only patients with frozen shoulder. 37.4% in Malaysia[37] and 32% in South Africa[38] among patients with chronic pain. And the study done in Adiss Abeba, 36.1%[22] and Wolayita Sodo, 36.4%[23] Ethiopia much lower than the present study. This dissimilarity might be the variant of study population or assessment tool, in this study we use Patient Health Questionnaire (PHQ-9) while the Malaysia study used Depression Anxiety Stress Scale-21 (DASS-21). The other studies also used Hospital Anxiety Depression Scale (HADS) and Beck Depression Inventory (BDI) assessment tool to screen depression for patients with musculoskeletal.

On the contrary, the burden seen in this study result was lower than the studies done in Liverpool, UK 72% [39]; include chronic pain, and used Structured Clinical Interview (SCID) diagnostic and Beck Depression Inventory (BDI) but in our study we use Patient Health Questionnaire (PHQ-9). 65.3% in Nigeria among general out patients [20] and also 71% in Saudi Arabia among patients with chronic pain [19]. This difference may be due to variation in study groups and might be different study design, sample size and sampling procedure.

The findings of this study shown that the duration of treatment is significantly associated with depression, with patients who had treatment durations of 3–4 weeks being more likely to have depression; treatment durations of more than 4 weeks increased the likelihood of having depression when compared to only having two weeks of treatment duration. This finding is supported by the study done in the UK [30]. The possible reason may be that musculoskeletal disorders require long-term treatment follow-up, which puts an economic burden (treatment cost) on the patient and may exhaust the patient [40].

Poor and moderate social support among musculoskeletal patients significantly increased the odds of depression compared to strong social support. This finding is in line with the study done in the UK in 2019 [41]. The possible explanation might be social support from family and friends plays an important role in lowering the likelihood of depressive symptoms and improving psychological quality of life [42].

## Conclusion

More than half the participants had positive symptoms of depression with PHQ-9 among musculoskeletal disorder patients treated in physiotherapy out-patient department. The length of hospital stays and the status of social support from families and friends were significantly associated with depression among patients with musculoskeletal disorders.

### Strength and limitation of the study

The strength of this study was the data was collected by using Amharic version validated Patient Health Questionnaire and conducted in multicenter this could increase the representativeness of the study. Even though it is the first study done in Ethiopia in depression among patients with musculoskeletal disorder, it has some limitation. The main limitation of this study was including small sample size and patients with musculoskeletal disorder were not assessed for a history of depression prior to the beginning of disorder. Secondly, history of use of antidepressant medications was not collected but almost all study participants had never been diagnosed for depression prior as part of routine care thus less likely to be in antidepressants medication. Thirdly, study’s cross-sectional design precluded a follow-up, which would have offered a better design for detecting characteristics linked to depression. The results were also relied on patients’ self- reported. This might have been subject to recall bias.

## Electronic supplementary material

Below is the link to the electronic supplementary material.

Supplementary Material 1 Informed consent statements

## Data Availability

The manuscript contains all of the data that is crucial to our findings. Requests for additional information on the dataset and questions about data sharing will be treated in accordance with a reasonable request to sermias131@gmail.com.

## List of Abbreviations

AOR: Adjusted odds ratio
BDI: Beck Depression Inventory
CI: Confidence interval
COD: Crude odds ratio
DASS-21: Depression Anxiety Stress Scale-21
DM: Diabetes Mellitus
Epi data: Epidemiological data
FHCSH: Felege Hiwot comprehensive specialized Hospital
G.C: Gregorian calendar
HADS: Hospital Anxiety Depression Scale
LMIC: lower and middle income country
LTSA: long term sickness absence
OPD: out-patient department
PHQ-9: patient health questioner-9
RVI: retroviral infection
SCID: Structured Clinical Interview diagnostic
SPSS: Statistical Package for Social Sciences
TGCSH: Tibebe Gihion comprehensive specialized
UK: united kingdom
UoGCSH: University of Gondar comprehensive specialized hospital
USA: United States of America
VAS: Visual analogue scale
WHO: world health organization
