# Supplementary material for: Magnitude of depression and associated risk factors among patients with musculoskeletal disorder treated in physiotherapy outpatient department in Amhara region comprehensive specialized hospital in Ethiopia: a prospective cross-sectional study
Source: BMC Psychiatry. 2023 Mar 22;23:189. doi: 10.1186/s12888-023-04658-3 (PMC10035282; doi:10.1186/s12888-023-04658-3)
Supplement: Supplementary file 1 — Supplementary Material 1 Informed consent statements [file 12888_2023_4658_MOESM1_ESM.docx]

**Informed Consent statements**

**University of Gondar College of Medicine and Health Sciences**

**Physiotherapy department**

**Title: Magnitude of depression and associated risk factors among patients with musculoskeletal disorder treated in Physiotherapy outpatient department.**

Good morning/afternoon, my name is ______ I am here on behalf of Ermias Solomon, Master of physiotherapy at University of Gondar College of Medicine and Health Sciences, Physiotherapy department. He has ethical clearance from the University of Gondar to research the above topic. We believe that the study findings will help to improve depression level in the future. If you participate in the study, it will not take more than 15-20 minutes. The information that you give using this questionnaire will be used only for research purpose and all information you provide to me will be strictly confidential. The study has no risk to you and your family members but mild time-consuming.

Therefore I politely request your cooperation to respond at all or to withdraw in the meantime, but your input has great value for the success of my objective.

**Certificate of consent**

I understand that the findings of this research will be disseminated to Hospital management and decision-makers that will be useful as an input for intervention design

I voluntarily consent to participate in this study.

I Agree Disagree

If you are agreeing to participate in the study please visit the next page.

If disagree, continue to the next participant by writing the reasons for refusal

Data collector: Name_______________________ Signature ___________

Supervisor: Name________________________ Signature__________

Date of interview______________________________

*Thank you for consenting to be a participant in this study*

For Data collectors use only:

Institution: _______________________ Questionnaire ID: ___________

**Section A: Demographic information**

**Please *indicate your opinion by ticking* 🗹 *the appropriate answer***

1. Age: ------------years old
2. Sex:
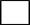
 male
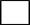
 female
3. Residence
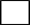
 Urban


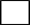
 Rural

1. Religion:
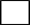
 Muslim
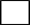
 Orthodox


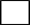
 Protestant
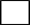
 Catholic
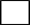
 Others (mention) ______

1. Educational status:
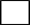
 not able to read and write
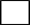
 able to read and write


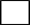
 Primary school
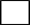
 Secondary school


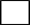
 College and above

1. Marital status:
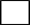
 single
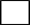
 married


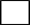
 Divorced
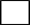
 Widowed

1. Occupation:
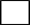
 Government employed
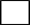
 Self-employed


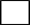
 House wife
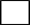
Unemployed
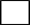
 Others______________

1. Medical Condition/physiotherapy diagnosis ___________________
2. How long received physiotherapy treatment __________________

**Section B clinical characteristics**

1. **Questions for Pain**

10. Do you have pain?


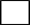
 Yes
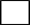
 B. No

- **If you say “yes” for question number 14, ask questions 15**
- **If you say “no” for question number 13, go to question number 16.**

11. How much pan do you experience?


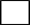
 No pain
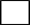
 mild pain
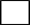
 moderate pain
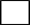
 severe pain

1. **Questions for Co-morbidity**

12. Do you have other medical condition?


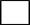
 Yes
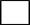
 no

- **If you say “yes” for question number 13, ask questions 17**
- **If you say “no” for question number 13, go to question number 18.**

13. What type of medical conditions?

___________________ ____________________ ____________________

**Section C: Question for social support**

The following three questions ask about how you experience your social relationships. The inquiry is about your immediate personal experiences. Please indicate the option that represents your experience.

| S.no | Social Support Questionnaire | Response |
| --- | --- | --- |
| 14 | How many people are so close to you that you can Count on them if you have serious personal problems (choose one option)? | 1.None  2.1 or 2  3.3-5  4.More than 5 |
| 15 | How much concern do people show in what you are doing (choose one option)? | 1. No concern and interest  2.Little concern and interest  3. Uncertain  4. some  5. a lot |
| 16 | How easy is it to get practical help from friends or dorm-mates’ if you should need it (choose one option)? | 5.Very easy  4. Easy  3. Possible  2. Difficult  1. Very difficult |

**Section D: Question for Depression** **patient health questioner-9 (PHQ-9)**

***Please indicate your opinion by ticking* 🗹 *the appropriate column***

|  |  | Not at all | Several days | More than half the day | Nearly every  Day |
| --- | --- | --- | --- | --- | --- |
| 17 | Little interest or pleasure in doing things |  |  |  |  |
| 18 | Feeling down, depressed, or hopeless |  |  |  |  |
| 19 | Trouble falling/staying asleep, sleeping too much |  |  |  |  |
| 20 | Feeling tired or having little energy |  |  |  |  |
| 21 | Poor appetite or overeating |  |  |  |  |
| 22 | Feeling bad about yourself or that you are a failure or  have let yourself or your family down |  |  |  |  |
| 23 | Trouble concentrating on things, such as reading the newspaper or watching television. |  |  |  |  |
| 24 | Moving or speaking so slowly that other people could have noticed. Or the opposite; being so fidgety or restless that you have been moving around a lot more than usual. |  |  |  |  |
| 25 | Thoughts that you would be better off dead or of hurting yourself in some way. |  |  |  |  |

*Thank you very much for your response!!*
